# Settlement of Planulae of the Moon Jellyfish *Aurelia aurita* onto Hydrophilic Polycarbonate Plates Modified by Atmospheric Plasma Treatment

**DOI:** 10.1371/journal.pone.0085569

**Published:** 2014-01-20

**Authors:** Akiko Tomaru, Ryota Sasaki, Hidekazu Miyahara, Akitoshi Okino, Nobuhiro Ogawa, Koji Hamasaki

**Affiliations:** 1 Department of Marine Ecosystem Dynamics, Atmosphere and Ocean Research Institute, The University of Tokyo, Kashiwa, Chiba, Japan; 2 Department of Energy Sciences, Tokyo Institute of Technology, Yokohama, Kanagawa, Japan; Harbin Institute of Technology, China

## Abstract

It has been reported that planula larvae of some jellyfish prefer artificial substrates for settlement. This research focused on the relationship between the settlement of planulae and the wettability of artificial substrate surfaces. We used atmospheric plasmas to change the wettability of the surfaces of polycarbonate (PC) plates because plasma treatment has no chemical side effects. The treatment made the surfaces hydrophilic, as evidenced by the decrease of contact angle from 85° to 35°. X-ray photoelectron spectroscopy revealed that the change of wettability of the PC plates could be attributed to N_2_, which was probably ionized in the air above the plates. Scanning electron microscopy revealed no difference in the surface morphology of the plates before and after plasma treatment. Results of bioassays using treated PC plates showed that planulae tended to preferentially settle on hydrophobic surfaces.

## Introduction

When jellyfish aggregate in great abundance and form blooms, there are significant environmental and economic impacts. Jellyfish in large numbers can compete with commercially valuable fish species for prey, consume larvae of commercially valuable fish, and break trawlers’ nets. In addition to their effects on fisheries, jellyfish can clog the intake pipes of the cooling systems of coastal power plants, sting and sometimes kill humans, and reduce tourism (reviewed in [1], [2], [3]). They disturb the ecosystem balance, for instance, by competing for food resources with zooplanktivorous fish, and the fact that they also consume fish eggs and larvae [4] might lead to the depletion of fish stocks (reviewed by [5], [6], [7], [8]).

The Moon jellyfish *Aurelia aurita* (Linnaeus) is a scyphozoan with a cosmopolitan distribution and is associated with widespread ecological and anthropological effects [9], [10], [11], [12]. During the 1960s, *A. aurita* blooms in Isefjord, Denmark, were a serious nuisance to the seine net and trap fisheries and caused damage by clogging the cooling-water intakes of coastal power plants [13]. In Japan, most Seto Inland Sea fishermen believe that the *A. aurita* populations have increased since the 1980s, and especially during the past 10 years [14]. Reports of jellyfish-related problems in Japan have undoubtedly increased in recent years [2]. Although *Aurelia* spp. are common jellyfish around the world, little is known about the factors that control their populations [15]. Research on scyphozoan blooms has concentrated on the ephyrae and adult medusae [2]. More recently, there has been recognition of the need to examine benthic stages. This research has centered on the sequence of steps from the settling of planulae through the growth of polyps and larval scyphistomae to the release of ephyrae by strobilation [15]. Polyps employ a variety of strategies to ensure survival. An important first step is the settling of planulae onto a suitable substrate. For this reason, we focused on the settlement of *A. aurita* (Linnaeus).

One of the reasons that it has been difficult to find *A. aurita* polyps in the field has been the paucity of information about their habitat. Settlement and metamorphosis of the polyps are controlled by a combination of many factors, including predation by benthic fauna, the location and physical characteristics of the substratum, contact with biofilms, and their gregarious behavior [16], [17], [18], [19], [20]. Polyps are found on a great variety of both living and nonliving natural substrata, including rocks, shells, polychaete tubes, ascidians, algae, and bryozoans. However, they also readily attach to artificial substrates, such as plastic or ceramic settling plates and many dock-building materials [11], [21], [22], [23], [24], [25], [26]. The construction of new structures within marine waters may increase polyp colony size [27].

In laboratory studies, several species of scyphozoans were recently found to prefer artificial to natural substrates [20], [28]. Brewer [29] studied the relationship between planula settlement and artificial substrates. He concluded that the planulae of *Cyanea* prefer the hydrophobic surfaces of both plastic and glass substrates. We tested the hypothesis that there was a relationship between settlement of *A. aurita* planulae and the wettability of a plastic surface. We chose plastic material for the settlement substrate because *A. labiate* planulae prefer a plastic substrate for settlement [30], and many species of invertebrates have been known to encrust plastic flotsam.

Plasma treatment involves the use of ionized gases to change the surface energy of a substrate and to produce a hydrophobic or hydrophilic surface. Plasma treatments are categorized on the basis of pressure, either low or atmospheric (e.g. [31]). Generally, low-pressure plasma treatment has been widely applied in materials processing, but low-pressure plasma treatment is associated with several disadvantages. Vacuum systems are expensive and require considerable maintenance. Furthermore, the size of the object that can be treated is limited by the size of the vacuum chamber. Atmospheric pressure plasma treatment overcomes the drawbacks of vacuum processes. In the early 1990s, studies of the applications of atmospheric plasma treatment gradually increased because of its low cost and flexibility for use in continuous processes [32]. In our atmospheric plasma treatment device (Atmospheric Damage-free Plasma, Linear; Plasma Concept Tokyo, Inc., Tokyo, Japan), the plasma-generating region was separate from the plasma-processing area. Use of this discrete plasma system avoided thermal/electric discharge damage to the target substrate and enabled treatment of not only dielectric (e.g. plastic or glass) but also electro-conductive materials (e.g. metal) [33]. A characteristic of our device is the fact that various gas species can be used: not only helium, argon, and oxygen, but also nitrogen, carbon dioxide, air, and so on [33]. To elucidate the changes in surface characteristics, we used X-ray photoelectron spectroscopy (XPS) to analyze the treated PC surfaces; and we used field emission scanning electric microscopy (FE-SEM) to observe the surface morphology of PC plates.

To avoid any chemical side effects and morphological changes of the substrate surface, we used atmospheric plasma treatment to change the hydrophobic surface of the PC plate to a hydrophilic surface. We then assayed the relative tendency of *A. aurita* planulae to settle on PC plates with different wettabilities.

## Materials and Methods

### Ethics Statement

No specific permits were required for the described field studies. The Mie prefecture local fishermen's union reviewed and approved our experimental protocol before we initiated sampling. Our studies of *A. aurita* did not involve endangered or protected species.

### Experiment 1. PC Plate Treatment

The PC plates were made from a commercially available plastic substrate. Polycarbonate consists of long-chain linear polyesters of carbonic acid and dihydricphenols, such as bisphenol A (–O–(C = O)–O– ). The PC plates (76×26×1 mm) were treated with an atmospheric plasma treatment device (Atmospheric Damage-free Plasma, Linear, 250 mm slit type; Plasma Concept Tokyo, Inc., Tokyo, Japan). The plasma gas consisted of Ar (30 L min^–1^) with O_2_, CO_2_ or N_2_ (0.1 L min^–1^). The PC plates were moved at a rate of 100 mm min^–1^ at a distance of 3 mm from the plasma source. We did not perform a pretreatment of plates before our plasma treatment. After plasma treatment of a PC plate, its wettability was quantified in terms of its contact angle, which was determined by the sessile drop method [34] with a contact angle goniometer (PG-X, MATSUBO Co., Tokyo Japan) and distilled water. All statistical analyses were done with the R 2.12.0 statistical software package (http://www.r-project.org/). The surface analyses of PC plates were performed with a PHI 5000 VersaProbe (ULVAC-PHI, INC., Chigasaki, Japan) XPS system. We used a micro-focused (100 µm, 25 W) Al K_α_ X-ray beam and a dual-beam charge neutralizer to compensate for the charge-up effect. We used an Ar ion gun for the sputter-etching (1-min sputtering time; 0.5-min interval; 3 cycles), which was done before the surface analysis. The envelopes of the C1s, N1s, and O1s peaks in the XPS spectra were fitted using MultiPak ver.9 (ULVAC-PHI) software. The surface morphological features of the PC plates were characterized with a model S-4800 (Hitachi High-Technologies Corporation, Tokyo, Japan) FE-SEM under an accelerating voltage of 1.0 kV. For the FE-SEM observations of prepared samples, PC plates were coated with carbon (ca. 5-nm thickness).

### Experiment 2. Planula Settlement on PC Plates

Sampling of *A. aurita* medusae was conducted during the daytime in Ise Bay, Japan, during November 2011. The sampling was carried out with a chartered ship. Female medusae with planula larvae were scooped from surface aggregations with a hand net (10-mm mesh size) or by SCUBA diving and kept in buckets with ambient seawater. Planula larvae were collected with a pipette from the brood sacs of the oral arms of ripe female medusae and were immediately transferred to glass bottles filled with ambient seawater. In the laboratory, the samples of planulae were washed with commercially purchased seawater (AORI seawater: seawater from offshore Hachijojima, Japan. Tokai Kisen Co., Ltd.), while being retained in a 25-µm mesh net. Planulae were immediately transferred to plastic bottles containing AORI seawater and kept at 5°C until the planula assay.

Control PC plate contact angles were 86.8±1.01°. In the plasma treatment contact angles were set at either 55° or 35° to enhance wettability. Plasma gas conditions were the same in Experiments 1 and 2. Table 1 summarizes the PC plates used in the planula assay.

**Table 1:** PC plate abbreviations.

Abbr	Plasma gas	Treated contact angle (°)
Cont85	No treatment	85
CO55	Ar with CO_2_	55
CO35		35
O55	Ar with O_2_	55
O35		35
N55	Ar with N_2_	55
N35		35

**Figure 1:**
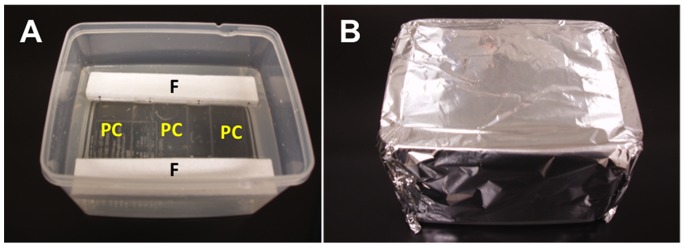
Experiment 2. PC plate planula assay. (A) PC plates (PC) were buoyed using floats (F). (B) The experimental containers were covered with aluminum foil.

The planulae of *A. aurita* (concentrations about 10 individuals mL^–1^) were suspended in AORI seawater. Both plasma-treated and untreated PC plates were buoyed using floats in the AORI seawater with the planulae in a plastic container (Figure 1A). Before the bioassay, the PC plates were not soaked in seawater, to avoid biofilm formation on the surface of the PC plates. The experimental containers were covered with aluminum foil to shield them from light (Figure 1B) and kept at 25°C. All the assays were carried out in triplicate. Settled planulae were counted using a stereomicroscope. The counting area was a 20×20 mm (1 grid) square on a counting sheet. The number of settled planulae was determined after 24 h.

All statistical analyses were carried out with the R 2.12.0 statistical software package (http://www.r-project.org/). Before the analysis of the planula settlement results, all data were square-root transformed [35].

## Results

### Experiment 1. Plasma Treatment

Table 2 shows the measured contact angles on both the untreated and plasma-treated PC plates. The contact angles of the plasma-treated PC plates were controlled at about 35° (CO35, O35, N35; contact angles in Table 2) for all the gas mixtures and were changed so as to be significantly smaller than the contact angles of the untreated PC plate (Cont85) just after treatment (Table 2) (One way ANOVA, *F* = 3079, df = 3, *p*<0.01; Tukey HSD, Cont85 vs CO35, O35, and N35, *p*<0.01). A decrease of the contact angle means that the wettability of the surface increased.

**Table 2:** The relationship between contact angle and plasma treatment.

Plasma gas	Treatment rate	Abbreviation	Contact angle (mean ± SD)		*t*-test	
	(mm sec^−1^)		Just after	After 24 hr	*t*-value	*p*-value
Ar with CO_2_	1	CO35	36.8±0.94	41.4±0.59	9.25	<0.01
	150	CO55	57.1±0.93	62.8±2.17	5.41	<0.01
Ar with O_2_	1	O35	34.0±1.44	43.4±1.01	11.98	<0.01
	150	O55	52.3±0.87	53.4±1.48	1.38	0.20
Ar with N_2_	1	N35	36.6±0.51	43.8±1.28	11.62	<0.01
	100	N55	58.0±0.80	61.9±0.38	10.01	<0.01
No treatment		Cont85	86.8±1.01		No test	

Results of *t*-test between before and after plasma treatment. *n* = 5, SD = standard deviation.

In Experiment 2 (the planula settlement bioassay), the contact angles of all treatments were controlled at about 55° (CO55, O55, and N55; contact angle in Table 2). The experiment revealed whether these contact angles remained constant or changed after 24 h of treatment under laboratory conditions. Except for O55, all contact angles increased significantly (*t*-test in Table 2). The planula settlement assay was carried out just after plasma treatment of the PC plates (Experiment 2). In addition, we confirmed that the wettability of the PC plates was constant for 5 days in both distilled water and AORI seawater (data not shown).

**Figure 2:**
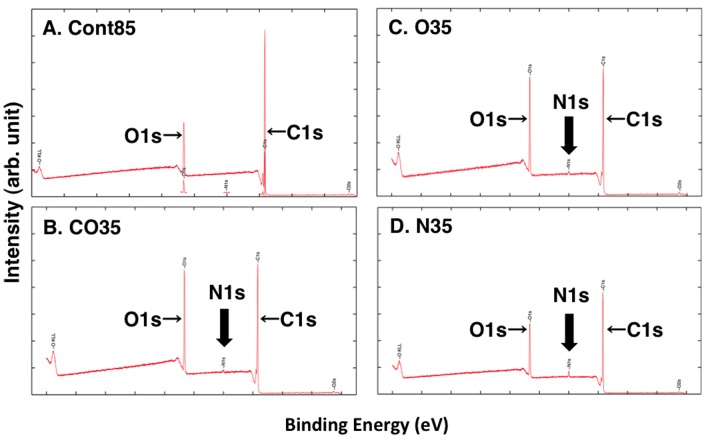
Wide-scan spectra of XPS analyses. (A) CONT, (B) CO35, (C) O35, (D) N35.

**Figure 3:**
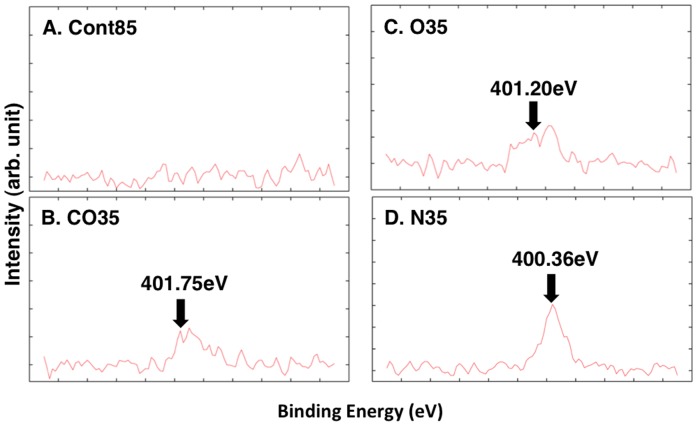
N1s spectra of XPS analyses. (A) CONT, (B) CO35, (C) O35, (D) N35.

XPS analyses were carried out to identify changes of the characteristics of the treated and untreated surfaces. Figure 2 shows wide-scan spectra of both the untreated (Cont85) and treated (CO35, O35, N35) PC plates. The intensity of the O1s peak was higher on the CO35 and O35 plates versus the Cont85 plate. An N1s peak was detected not only on the N35 plate but also on the CO35 and O35 plates; however, the N1s peak was not detected on the Cont85 plate. Figure 3 shows the XPS spectra of the C1s and O1s regions of the untreated and plasma-treated PC plate surfaces. The N1s spectrum shows that the peak has binding energies of 401.20 eV, 401.75 eV, and 400.36 eV on the CO35, O35, and N35 plates, respectively.

**Figure 4:**
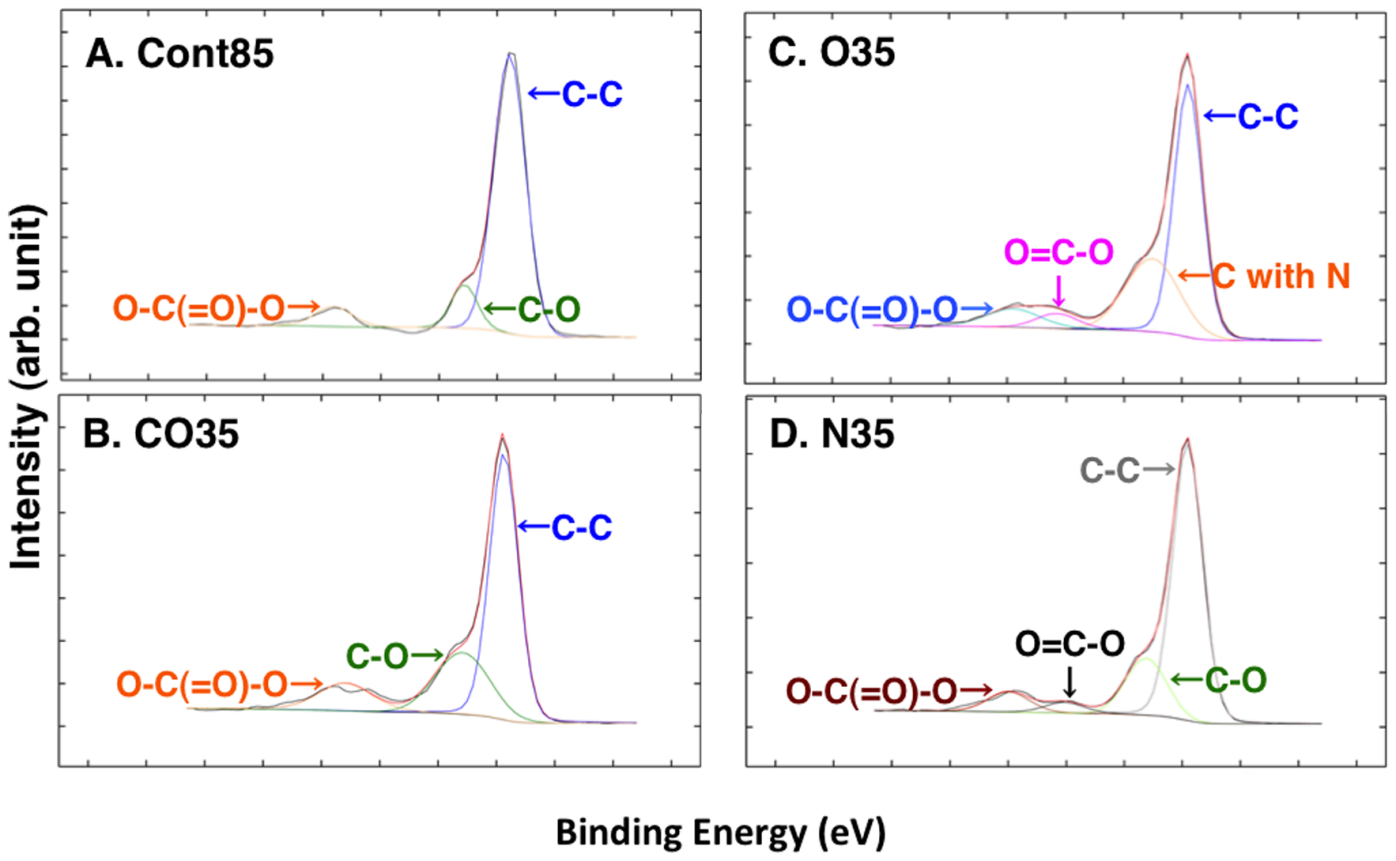
C1s spectra of XPS analyses. (A) CONT, (B) CO35, (C) O35, (D) N35.

**Figure 5:**
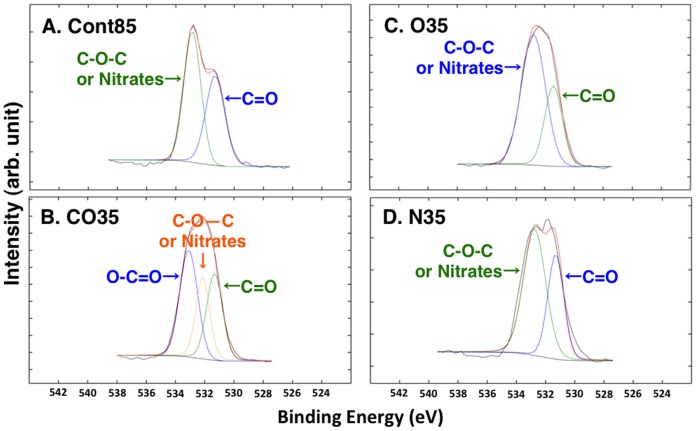
O1s spectra of XPS analyses. (A) CONT, (B) CO35, (C) O35, (D) N35.

The XPS spectra of the untreated and plasma-treated PC plate surfaces are shown in Figure 4 and Table 3 for the C1S region and in Figure 5 and Table 4 for the O1s region. The C1s spectrum of the plasma-treated PC plates revealed compounds that were different from those on the Cont85 plate. In the case of the CO35 plate (Figure 4B), although the compounds were the same as those on the Cont85 plate (Figure 4A), the –(C–O)–, and –(O = C–O)– peaks, normalized to the sum of their intensities, were larger (Table 3). On the O35 plate (Figure 4C) and the N35 plate (Figure 4D) carbon-nitrogen and –(O = C–O)– moieties were detected instead of the –(C–O)– moiety on the Cont85 plate (Table 3). The chemical moieties identified by the O1s and C1s spectra were the same. The O1s spectrum implied the presence of similar moieties on both the Cont85 plate and the plasma-treated PC plates. The same moieties were detected on the O35, N35, and Cont85 plates, but an additional moiety was detected on the CO35 plate. Although the O1s spectra of both the O35-plate (Figure 5C) and the N35 plate (Figure 5D) were similar to the O1s spectrum of the Cont85 plate (Figure 5A), the –(C–O–C)– and/or nitrate binding energies of both the O35-plate and the N35 plate were higher than those of the Cont85 plate (Table 4). The –(O–C = O)– moiety was detected on only the CO35 plate (Figure 5B) (Table 4).

**Table 3:** The peaks (eV) and areas (%) of the C1s spectra from the XPS analyses.

Compound	Cont85		CO35		O35		N35	
	Peak (eV)	Area (%)	Peak (eV)	Area (%)	Peak (eV)	Area (%)	Peak (eV)	Area (%)
C-C	284.60	82.40	284.60	61.71	284.60	56.62	284.60	70.21
C with N	N.D.	N.D.	N.D.	N.D.	285.83	30	286.03	18.77
C-O	286.16	10.54	286.02	25.69	N.D.	N.D.	N.D.	N.D.
O = C-O	N.D.	N.D.	N.D.	N.D.	289.12	4.95	288.84	3.86
O-C( = O)-O	290.65	7.06	290.05	12.60	290.62	8.43	290.76	7.16

N.D., not detected.

**Table 4:** The peaks (eV) and areas (%) of the O1s spectra from the XPS analyses.

Compound	Cont85		CO35		O35		N35	
	Peak (eV)	Area (%)	Peak (eV)	Area (%)	Peak (eV)	Area (%)	Peak (eV)	Area (%)
C = O	532.33	44.46	532.20	31.61	532.22	31.87	532.09	37.23
C-O-C or Nitrates	533.88	55.54	533.00	26.79	533.60	68.13	533.53	62.77
O-C = O	N.D.	N.D.	533.97	41.60	N.D.	N.D.	N.D.	N.D.

N.D., not detected.

**Figure 6:**
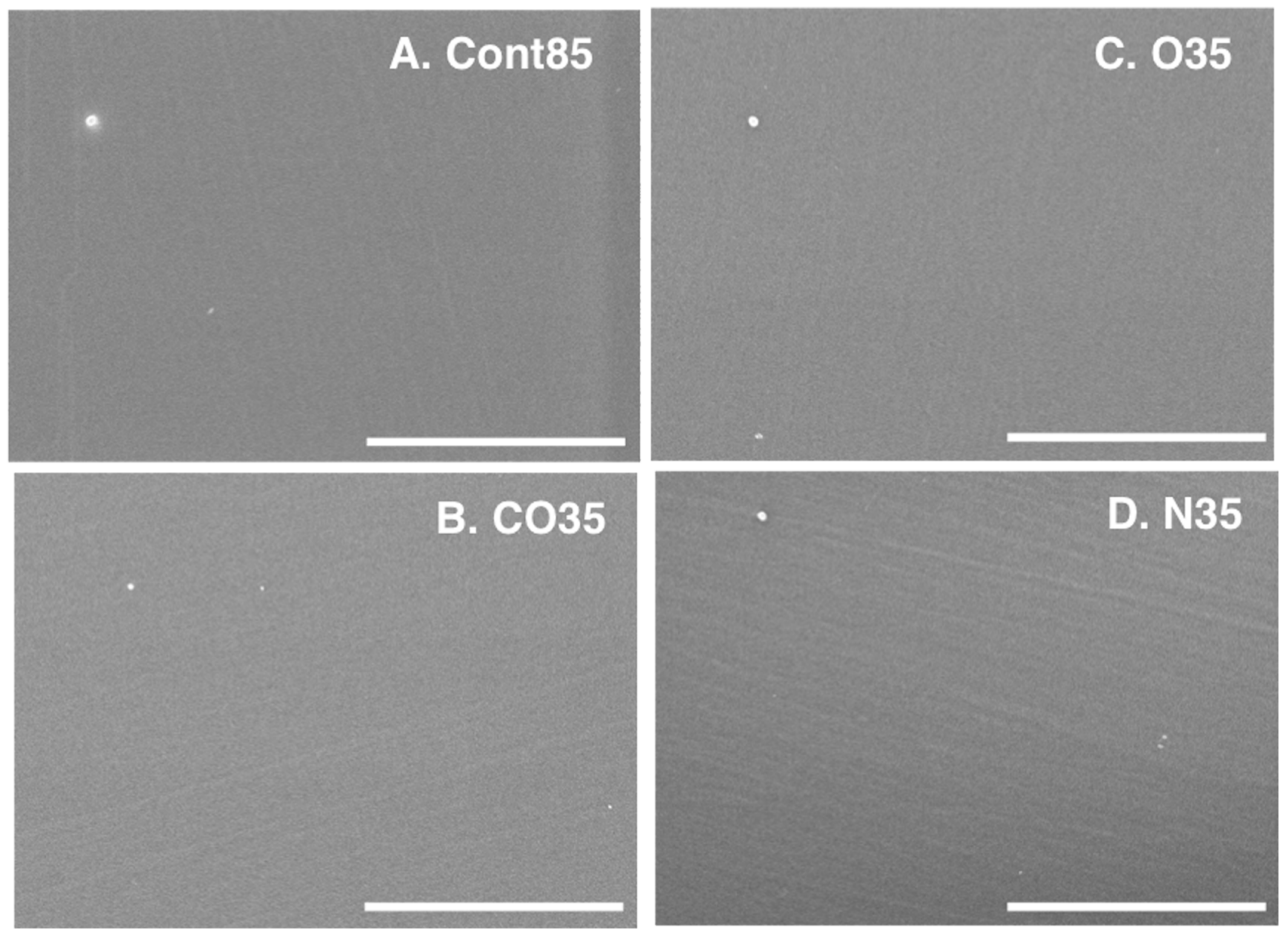
Typical FE-SEM images. (A) Cont85, (B) CO35, (C) O35*, (D) N35. Bar = 5 µm. *: Ar (30 L min^–1^) with O_2_ (0.02 L min^–1^).

Figure 6 shows typical SEM images of the untreated and plasma-treated surfaces of PC plates. Figure 6A is a typical image of an untreated PC plate. The white round object on the PC plate is a dust particle. The surface structure of both the plasma-treated and untreated PC plates was flat, with no apparent differences.

### Experiment 2. PC Plate Planula Assays

**Figure 7:**
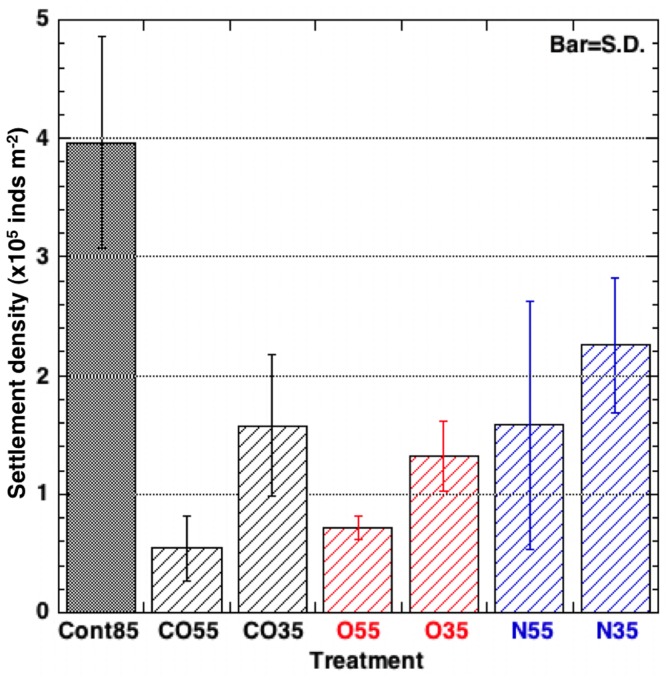
The relationship between contact angle and settlement of planulae. Refer to Table 1 for sample abbreviations.

The surface wettability of the PC plates affected planula settlement (Figure 7). The planulae showed a greater tendency to settle on a hydrophobic surface than on a hydrophilic surface (one-way ANOVA: df = 6, *F* = 10.32, *p*<0.01; Tukey HSD test: differences of settlement on the Cont85 plate vs treatment plates, expect for Cont85 vs N35, were significant at *p*<0.01). However, the extent of planula settlement when the contact angle was 35° was greater than when the contact angle was 55°. Therefore, the extent of planula settlement was not in proportion to the contact angle.

## Discussion

### Experiment 1. Plasma Treatment

Our results indicate that there was a nitrogen group on the surface of all the treated PC plates (CO35, O35, and N35). No nitrogen group was detected on the untreated PC plate (Cont85 in Figs. 2 and 3). Because the plasma-generating region of our atmospheric plasma treatment device was separate from the plasma-processing area, some air was therefore present during plasma generation, and N_2_ in the air was ionized. Consequently, a nitrogen group was detected in the spectra of CO35 and O35 (Figs. 4 and 5). The nitrogen group on the PC plates may have contributed to a change of wettability. Other additional chemical moieties were revealed by the XPS data. The treatment introduced new polar groups on the surface, and the relative contribution of –(C–C)– groups decreased, especially in the C1s spectrum (Table 3). Those results indicate that some of the carbon bonds on the PC surface were broken and subsequently reacted with oxygen gas, the result being formation of oxygen-containing compounds. It is generally accepted that the new oxygen-containing groups that form on polymer surfaces during oxidation processes contribute fundamentally to the enhancement of surface hydrophilicity (e.g. [36], [37], [38], [39], [40], [41]). In a polymer, it is well known that increases of surface energy and changes in the wetting characteristics of the surface from hydrophobic to hydrophilic can be attributed to insertion of new polar groups [42]. Our results are consistent with the results of Yaghoubi and Taghavinia [41], who used compressed air, but we have also demonstrated changes in wetting characteristics with CO_2_ and N_2_. SEM images showed that both the plasma-treated and untreated PC plates had similar morphologies (Figure 6). This conclusion is supported by previous reports that plasma-treated and untreated PC surfaces show similar morphologies in observations made by SEM [38] and atomic force microscopy (AFM) [41].

### Experiment 2. PC Plate Planula Assays

Planulae of *A. aurita* showed a tendency to settle on hydrophobic surfaces. However, no relationship was apparent in our results between the contact angle of the PC plates and the tendency of planulae to settle. Although our results showed that settlement was lower at 55° than at 35° for the same gas mixture in all gas treatments, the difference in settlement was not significant (Tukey HSD test). Hence our results do not imply that settlement is minimal at some angle intermediate between 35° and 85°. One of the classical explanations for the relationship between the settlement of Bryozoan larvae and wettability (hydrophobicity) has been surface free energy (e.g. [43]). Plasma treatment uses ionized gases to change the surface energy. However, our results did not reveal a relationship between the number of planulae that settled and the contact angle of the PC plates. Perhaps settlement cannot be explained solely on the basis of the surface free energy of the PC plate. Brewer [29], however, showed a correlation between the number of *Cyanea* planulae that attached to surfaces and the surface wettability; the correlation strongly suggested that there was a relationship between *Cyanea* planulae attachment and surface wettability. He concluded that an explanation based entirely on surface free energy was an incomplete explanation of the interaction between planulae and hydrophobic surfaces. He pointed out that the attachment of *Cyanea* planulae is influenced by many factors, such as duration of inspection, time required to attach, and size of encysted planulae.

Holst and Jarms [20] pointed out the important role of artificial substrates (concrete, machined wood, polyethylene, and glass) for planula settlement of five scyphozoan species. Moreover, Hoover and Purcell [15] reported that plastic (polyethylene) was the preferred substrate for settlement of *A. labiate* compared to other dock-building materials (wood and rubber). A previous report [44] has pointed out that many species of invertebrates encrust plastic flotsam, and the abundance and distribution of these organisms may be increasing. At least during the 1970s, the amount of plastic litter increased enormously worldwide (e.g. [45]). Our study implies that increasing amounts of plastic flotsam may be one of the causes of the expansion of the habitat of many species of jellyfish.

Holst and Jarms [20] and Hoover and Purcell [30] found that planulae may preferentially settle on biofilms; their experimental methods involved growth of bacterial films on substrate plates before the assay [20], and their observations extended over a period of 4 weeks [30]. We hypothesize that plastic flotsam, regardless of the amount of time it has been exposed to seawater, is a potential habitat for jellyfish polyps and a vector for transporting jellyfish to new habitats. Lo et al. [27] concluded that increases in the prevalence of floating structures probably leads to an expansion of habitats favorable for jellyfish polyps and locally to an increase of jellyfish populations, especially in areas where water flow is restricted. The implication is that construction of submarine structures and the increasing presence of marine litter are both factors that have contributed to the increase of jellyfish biomass because they enhance survival of polyps. An increase in the survival of polyps has a high potential to lead to blooms of jellyfish.
